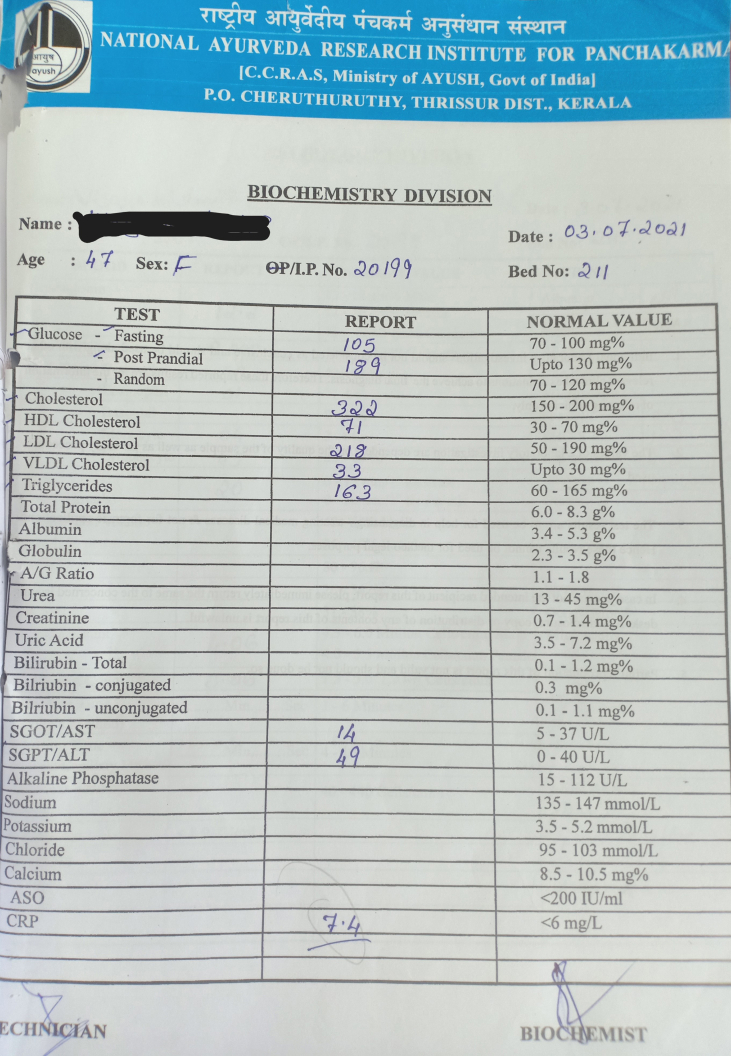# Ayurvedic management of neurological deficits post COVID-19 vaccination - A report of two cases

**DOI:** 10.1016/j.jaim.2023.100737

**Published:** 2023-06-08

**Authors:** K.M. Pratap Shankar, P. Nair Pratibha, V. Saritha

**Affiliations:** a National Ayurveda Research Institute for Panchakarma, Cheruthuruthy, Thrissur, Kerala, India; b Department Of Kayachikitsa, VPSV Ayurveda College, Kottakkal, Kerala, India; c Department of Radiology, Government Medical College, Palakkad, Kerala, India

**Keywords:** COVID-19 vaccination, Acute disseminated encephalomyelitis, Multiple sclerosis, Ayurveda

## Abstract

The world witnessed much research fund allocation on the COVID-19 outbreak's epidemiology, pathology, impact on lifestyles, social behaviours and treatment possibilities. The highly contagious nature of the disease compelled scientific communities and related organisations to hasten vaccine development and supplies. Well-timed international collaborations resulted in quicker development of varied forms of vaccines against COVID-19. Prospective observational studies and systematic reviews on vaccine trials reported their safety and efficacies. Nevertheless, post-marketing surveillance is quintessential to ascertain such safety and efficacy claims. There have been scattered reports lately of several adverse temporal events, such as haematological, immunological and neurological untoward occurrences following COVID-19 inoculation. There is a growing piece of evidence of the impact of COVID vaccination on patients with neurological-neuroimmunological disorders. Here two unrelated cases of neurological deficits post-COVID vaccination are reported. One was an incidence of Acute Disseminated Encephalomyelitis, while the other was an acute exacerbation of Multiple Sclerosis following vaccination. Ayurvedic treatments were effective in either of these conditions. Case series and case reports shall judiciously add information to vaccine safety data and acknowledge the necessity of clinician approval, based on detailed individualised assessments before mass vaccination.

## 1 Introduction

Acute disseminated encephalomyelitis (ADEM) and Multiple sclerosis (MS) are categorically grouped under the shared pathogenesis of defective immune-mediated responses in the form of inflammation-driven demyelinating conditions [1]. ADEM is usually referred to as a ‘post-infectious encephalomyelitis', which stands for an acute and rapidly progressing autoimmune response in CNS characterised by inflammation and demyelination essentially following an infection or immunisation [2]. MS on the other hand portrays chronic inflammation followed by demyelination, gliosis and neural loss wherein clinical symptoms range from an acute and variable manifestation of vision impairment, cognitive dysfunction, focal weakness, and bladder and bowel incontinence to relapsing-remitting and progressive forms that may later advance to permanent disability [3].

Autoimmune demyelinating disorders result from defective immune responses at varied levels resulting in multifocal neurological symptoms. Thus, clinical judgment and subsequent selective management become challenging, which might result in more comprehensive therapeutic strategies, prolonged hospital stays, and supportive care to prevent progressive disability. Though primary antiviral management is advocated in ADEM presenting with evident acute inflammatory signs and symptoms of encephalopathy, the strategic treatment is immunosuppression with glucocorticoids, immune globulins, and cyclophosphamides or plasma exchange [2].

Disease-modifying drugs (DMDs) are commonly prescribed in MS such as Glatiramer acetate, Interferon-beta molecules, Natalizumab, Mitoxantrone, and Fingolimod which act differently against respective pathological processes at different sites [3]. The fact remains that these disease-modifying agents are primarily indicated in primary inflammation-driven phases of the disease and are found less effective in secondary degenerative phases. Also, long-term therapy with such DMDs may be needed to prevent relapses or permanent disability.

Though COVID-19 vaccine safety in individuals diagnosed with chronic autoimmune diseases is not established, on account of random and unpredictable events noticed with SARS-COV-2 affliction (significantly contributing to disease morbidity); a vaccination drive is recommended to all such groups. Circumstantial pieces of evidence lead to an association between COVID-19 vaccination and nervous system inflammation [4].

Few case reports are made public on neurological complications of the COVID-19 vaccination [[5], [6], [7], [8], [9]]. Discussions are underway concerning caution while administering the COVID-19 vaccine in people with pre-existing inflammatory neurological conditions especially those on disease modifiers that suppress the immune mechanisms [10,11]. Acute relapse of autoimmune neurological conditions such as MS following COVID-19 vaccination is reported [12]. Also, it may be reasonable to prioritise their vaccination against COVID-19, advising them of receiving booster doses and the continuation of protective measures, even after being vaccinated. Riccardo Nistri et al., 2021 discussed the plausibility of the COVID-19 vaccination causing a relapse of MS with 16 cases [5].

Here are two such cases of post-ChAdOx19 (Recombinant) vaccination. The first case is an ADEM; the second is a progressive relapsing MS. Both cases experienced an abrupt manifestation of classic neurological symptoms indicative of the above-cited conditions. Herbal therapies and traditional medicine are increasingly being proposed for autoimmune conditions [[13], [14], [15], [16], [17], [18], [19], [20]]. Ayurvedic Science stands for traditional and complementary medical systems in India. Ayurvedic physicians consider neurological and associated categories of disorders under the shared pathological processes of ‘Vatavyadhi’ wherein there is a fundamental disruption in ‘Vatadosha’, one of the three functionally dynamic entities causally associated with health and disease. Further, the symptomatology of MS and ADEM noticeably simulates specific established pathological presentations mentioned in Ayurvedic textbooks such as Vatarakta, Avaritavata, Sannipatajwara, Dhathugatajwara, and Dhathukshaya. These two cases reported here were managed with Ayurvedic medicines and treatment procedures.

### 1.1 Case 01

#### 1.1.1 Patient information & diagnostic assessment

A 49-year-old healthy woman with no known history of diabetes mellitus, hypertension or other systemic diseases received her first ChAdOx19 (Recombinant) vaccination [COVISHIELD] on the 6th of March, 2021. The same day the patient developed muscle fatigue in both upper limbs, low backache, and pain in the posterior aspect of the right knee. The next day her low backache worsened and she developed cramps in her right calf muscles. Also, there was throbbing pain at the site of vaccination. The following day, symptoms persisted and her sleep was significantly disturbed. The patient visited the Institute and was under medication for the above-cited symptoms. She was advised to take Rasnasaptakam Kashayam (15 ml twice daily), Yogaraja Guggulu (one tab twice daily after food) & Triphala choornam (two tsp bed time) and was prescribed with Kottamchukkadi Thylam for external application. Symptoms gradually reduced but persisted until the following month, wherein she received her second dose of vaccine i.e. on the 3rd of April. All the symptoms aggravated and again she visited the Out Patient Department. On the 19th of April, the patient developed paresthesia in her right upper & lower extremities, low back pain, and cramps in her right leg that aggravated contact with cold water. Also, she felt a loss of temperature sensation in her right leg. She consulted a nearby Orthopaedic clinic following which an MRI of the lumbosacral spine revealed mild loss of lumbar lordosis and multilevel disc desiccation changes. The patient received analgesics.

On the 2nd of May, she developed weakness in her right lower limb. The next day she noticed weakness in her right hand as she had difficulty writing and a significant change in her handwriting, associated with recent memory loss, dyscalculia, and dysarthria. The same day the patient consulted a Neuro-physician who advised a brain MRI. She underwent an MRI scan where multiple enhancing lesions in the bilateral cerebral hemisphere with the possibility of neoplasm or tumefactive demyelination were detected [Supl file 1 A& 1 B]. The patient underwent stereotactic/open biopsy and received IV Mannitol 100 ml BD & IV Levipil 500 BD for three days. Then she was referred to a higher centre for further evaluation. Symptoms progressed. On the 6th of May contrast MRI brain imaging was suggestive of demyelination. MRI with spectroscopy and percussion study noted varying sized peripheral rim enhancing with central T2 hyperintense signal & peripheral intermediate signal at bilateral posterior temporal, right centrum semiovale, left corona radiate & in the right sub-lentiform internal capsule extending into the right crus. The imaging features indicated tumefactive demyelination (tumefactive ADEM) [Fig. 1]. ANA test was negative and the CSF study was normal. CSF for NMOSD was negative for NMO and MOG antibodies [Supl file 2]. MS evaluation panel ruled out the possibility of Multiple sclerosis. She received IV methylprednisolone and a short course of oral steroids. There was a notable improvement in weakness and she could walk without support. She was discharged with advice to continue oral methylprednisolone for seven days.

**Fig. 1:**
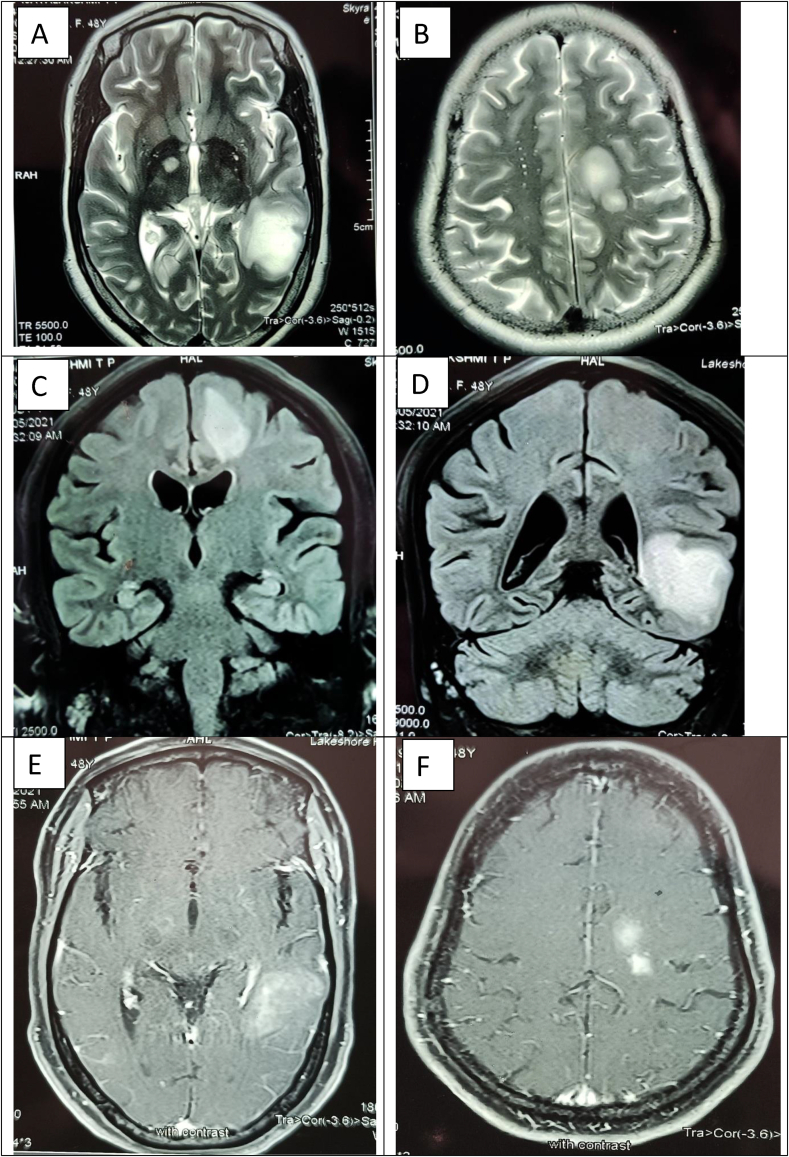
Brain magnetic resonance imaging (MRI) findings (Pre-treatment findings). Varying-sized peripheral rim-enhancing lesions with central T2 hyperintense signal & peripheral intermediate signal were noted in the bilateral posterior temporal, right centrum semiovale, left corona radiata, and the sub-lentiform internal capsule extending. The most significant lesion in the left posterior temporal lobe measures 3.4 x 3.0 cm. Minimal perilesional oedema was noted around this lesion with an open ring enhancement (open towards the cortex). The rest of the lesions show complete ring/heterogenous enhancement. Peripheral diffusion restriction was noted in the lesions with mild (A & B: axial view, C & D: coronal section, E & F – Post-contrast axial view).

On the 4th of June, right-sided weakness and dysarthria worsened which became evident after stopping steroids. She was again admitted and treated with IV methylprednisolone (IVMP) for five days. There was a marked improvement in weakness following which she was discharged with medical advice on tapering steroids. On the 11th of June, the patient visited Out Patient Department at NARIP presenting with right-sided weakness, unsteady gait, paraesthesia in lower limbs, emotional disturbances, low backache, intentional tremors (exacerbating on holding things), and postural tremors (more on the extension of the elbow joint) in the right upper limb.

#### 1.1.2 Clinical findings

On the 2nd of July, the patient was admitted to the Institute for rehabilitative care. Neurological examination revealed hemiataxic gait, power of 4/5 in the right upper limb and 3/5 in the right lower limb, reduced cutaneous sensations of pain, touch & vibration, and reduced joint proprioception in the right upper & lower limbs. The scale for assessing and rating the ataxia [SARA] score was 17. Blood reports revealed a mild rise in CRP levels (7.4 mg/L) and postprandial blood sugar levels (189 mg%) [Supl file 3].

#### 1.1.3 Timeline

Fig. 2 corresponds to the graphical representation of the clinical course of the condition in the patient and the effectiveness of Ayurvedic management as an add-on advocacy in managing clinical symptomatology. On the same day as the first dose of Covid-19 vaccination, the patient experienced muscle fatigue & low backache which gradually reduced with Ayurvedic internal medications. A few days after the second vaccination dose, all symptoms exacerbated. Also, paresthesia in her right upper & lower extremities manifested.

**Fig. 2:**
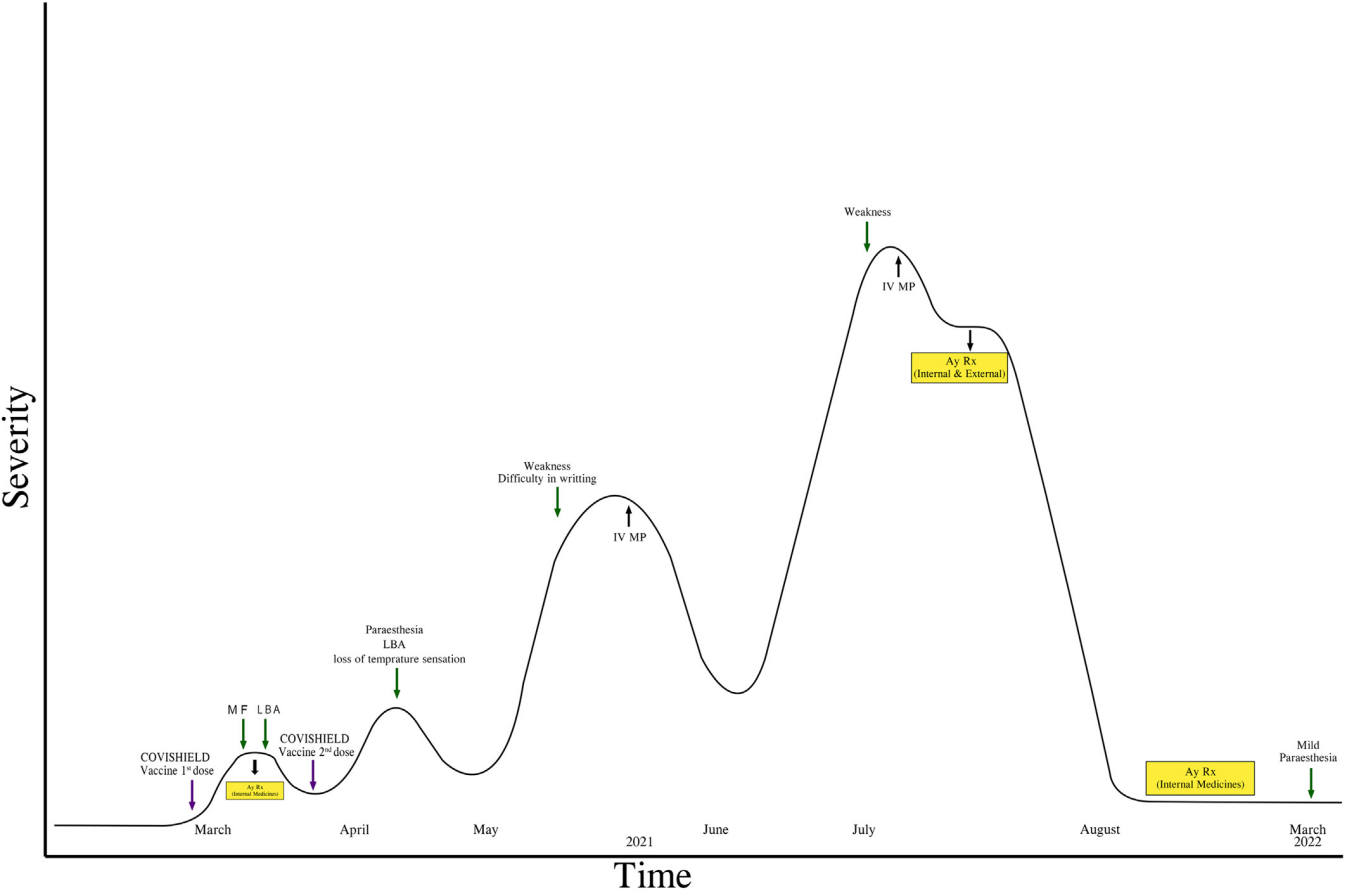
Clinical course of the condition of Case 01 demonstrating the severity of symptoms developed after two doses of Covid-19 vaccination. The symptoms evolved after the first vaccination, aggravated after the second dose and steadily escalated in a few months with a moderate decrease while on IV-MP. Further, it can be inferred from the graph that there was a deceleration in the symptoms after administering Ayurvedic treatments, and the condition remained almost normal during the follow-up period. Abbreviations: MF, Muscle Fatigue; LBA, Low Back Ache; IV-MP, Intravenous Methylprednisolone; Ay Rx, Ayurvedic treatments.

These symptoms gradually aggravated and she received IV methylprednisolone and a short course of oral steroids. On withdrawal from steroids, she developed right-sided weakness and dysarthria. IV methylprednisolone was administered for five days and there was a notable improvement in weakness.

Further, as the condition persisted she was admitted and underwent rehabilitative care at the Institute. She responded well after a month-long treatment comprising Ayurvedic internal medications and external treatments. Further, after discharge, she was advised of Ayurvedic internal medications. From the graph, it is noticeable that the patient's clinical status remained almost normal during the follow-up period of eight months.

#### 1.1.4 Therapeutic intervention

The patient underwent Ayurvedic treatments comprising internal medications and external therapies until the 5th of August [Table 1, Table 2]. Oral Steroids on tapering doses and anti-diabetic medicine continued during this period.

**Table 1:** Details of Internal medications with dosage and duration of Cases 01 & 02.

S.No.	Internal medications	Dosage	Date	Mode of action
**Case 01**				
1.	Amruthotharam Kashaya	15 ml BD	02/07/2021–24/07/2021	Jwaraharam & pachana property
2.	Ekanga vira ras Vati	One BD	02/07/2021–24/07/2021	Vata kapha haram
3.	Nishamlaki Choornam	One tsp BD	02/07/2021–05/08/2021	Anti diabetic
4.	Gorochanadi Gulika	One TID	02/07/2021–24/07/2021	Vata kapha haram & Jwara haram
5.	Sunti dhanyakam paneeyam	As paneeyam	02/07/2021–05/08/2021	Pachanam
6.	Guduchi Satvam	One pinch with Kashaya	02/07/2021–05/08/2021	Jwaraharam
7.	Rasnerandadi Kashayam	15 ml BD	25/07/2021–05/08/2021	Vatarakta haram
8.	Gopichandanadi Gulika	One TID	25/07/2021–05/08/2021	Vata pitta haram & jwaraharam
Discharge medicines from 06/08/2021				
9.	Rasnerandadi Kashayam	15 ml BD	One month	Vatarakta haram
10.	Mahamanjistadi Kashayam	15 ml BD	One month	Vatarakta haram
11.	Guduchi Satvam	One pinch with Kashaya	One month	Vatarakta haram
12.	Gopichandanadi Gulika	One TID	One month	Vata pitta haram & jwaraharam
Follow-up medicines from 09/09/2021				
13.	Mahamanjistadi Kashayam	15 ml BD	Three months	Vatarakta haram
14.	Kaisora guggulu	One BD	Three months	Vatarakta haram
**Case 02**				
1.	Mahamanjistadi Kashayam	15 ml BD	27/12/2021–13/01/2021	Vatarakta haram
2.	Amruthotharam Kashayam	15 ml BD	27/12/2021–13/01/2021	Jwaraharam & Pachanam
3.	Sanjivani vati	One BD	27/12/2021–13/01/2021	Jwaraharam & Pachanam
4.	Triphala choornam	10 gms HS	27/12/2021–13/01/2021	Vataanulomana
Discharge medicines				
5.	Mahamanjistadi Kashayam	15 ml BD	From 14/01/2021 -Three months	Vatarakta haram
6.	Kaisora guggulu	One BD	From 14/01/2021 -Three months	Vatarakta haram
7.	Chyavanaprasham	10 gms HS	From 14/01/2021 -Three months	Rasayanam
8.	Ksheerabala 101 thylam	15 drops with kashayam	From 14/01/2021 -Three months	Vatahara, Vata rakta hara

**Table 2:** Details of external procedures, drugs used and duration (in days) for Cases 01 & 02.

S.No.	Time period	Procedure	Drugs used	Quantity	Days	Mode of action
**Case 01**						
1.	02/07/2021–04/07/2021	Udvartanam	Triphala Choornam	Q.S	Three	Rooksha chikitsa
2.	05/07/2021–18/07/2021	Dhanyamla Dhara	Dhanyamla	Q.S	14	
3.	19/07/2021–25/07/2021	Guluchyadi Yapana vasti	Guluchyadi Kashayam	400 ml	Seven	Vatarakta hara
			4. Madhuyastyadi thylam	100 ml		
			5. Tiktaka ghritam	100 ml		
			6. Satahwa kalka	30 gms		
			7. Saindhava lavanam	15 gms		
			8. Honey	200 ml		
9.	26/07/2021–30/07/2021	Abhyanga	Dasamoola bala thylam	Q.S	Five	Vyana vata hara
10.	31/07/2021–06/07/2021	Pizhichil	Dasamoola bala thylam	3 ltrs	Seven	
11.	31/07/2021–06/08/2021	Thalam	Rasnadi choornam and Ksheerabala thylam	Q.S	Three	Prana udana vata hara
12.	31/07/2021–06/08/2021	Siro Pichu	Bala thylam	Q.S	Four	
**Case 02**						
1.	29/11/2021–05/12/2021	Udvartanam	Yava kulattha choornam	Q.S	Seven	Rooksha chikitsa
2.	06/12/2021–12/12/2021	Abhyangam	Sahacharadi thylam	Q.S	Seven	Vyana vata hara
3.	06/12/2021–12/12/2021	Ksheera kashaya dhara	Vidaryadi Ksheera kashaya	Q.S	Seven	Vatarakta hara
4.	11/12/2021–15/12/2021	Matra vasti	Sahacharadi Mezhupakam tailam	30 ml	Five	Vatarakta hara Rasayana
5.	13/12/2021–15/12/2021	Mustadi Rajayapana vasti	Makshikam (Honey)	200 ml	Five	
			6. Lavanam	15 gms		
			7. Sneham: Ksheerabala Thylam Sukumara ghritam	200 ml		
			8. Kalkam: Satapushpa, Madhuka, Kutaja, Rasanjanam and Priyangu	30 gms		
			9. Ksheera kashayam: Musta, Pata, Guduchi, Erandam, Bala, Rasna, Punarnava, Manjistha, Aragwada, Usira, Trayamana, Aksha, Rohin, Laghu panchamoola and Madanaphala	400 ml		
			10. Mamsa rasam	100 ml		
11.	24/12/2021–30/12/2021	Nasyam	Ksheerabala thylam	1.5 ml	Seven	Prana udana vata hara
12.	31/12/2021–06/01/2022	Shirodhara	Ksheerabala thylam		Seven	
13.	07/01/2022–13/01/2022	Annalepanam	Sastika choornam, balamoola kwatha and milk		Seven	Sthairyakrith and Bruhmanam

#### 1.1.5 Follow-up and outcomes

**Fig. 3:**
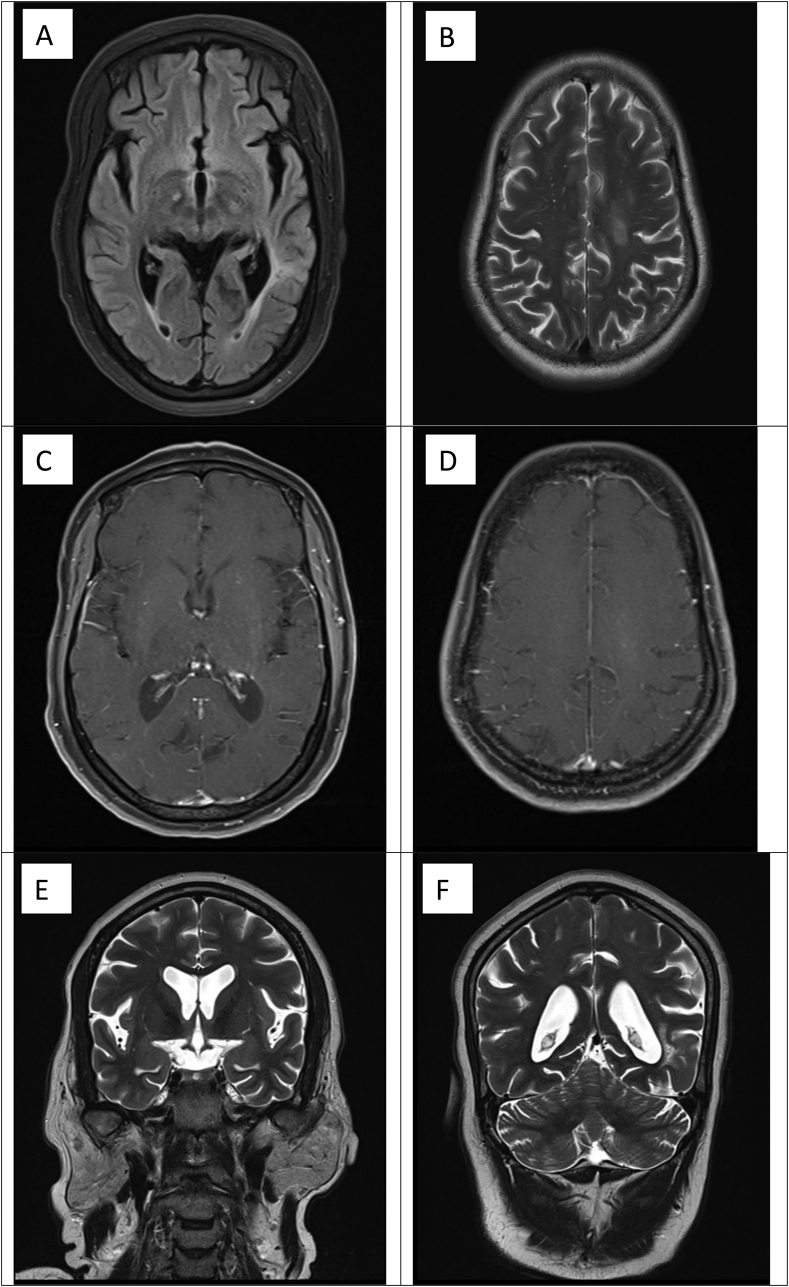
Brain magnetic resonance imaging (MRI) findings (Follow up). Confluent T2 and FLAIR hyperintensity in left centrum semioval, left posterior temporal, parietal deep and periventricular white matter. Faint enhancement in post-contrast. Non-enhancing, FLAIR hyperintense white matter foci in right frontal, parietal and occipital lobes and right & left sub lentiform internal capsule. No new lesions were noted in the follow-up study. Abbreviations: FLAIR, Fluid attenuated inversion recovery.

While in treatment, weakness gradually reduced with a notable reduction in paresthesia, low backache, and tremors. Speech turned out to be normal. The patient could walk without support (SARA gait score 0). At discharge and during the follow-up period, she continued Ayurvedic internal medications. During a follow-up check-up after eight months, she had mild paraesthesia in the lower 1/3rd of both lower limbs and no other neurological deficits. A follow-up MRI brain done on the 23rd of March 2022 noted no new lesions and considerable reduction in old lesions with faint enhancement post-contrast [Fig. 3].

### 1.2 Case 02

#### 1.2.1 Patient information & diagnostic assessment

A 39-year-old woman developed paraesthesia below the umbilicus and in both lower limbs in April 2017. The symptoms persisted for ten days and subsided without any treatment. Later, in 2019 she experienced blurred vision with difficulties in reading and mild bladder & faecal incontinence. These symptoms resolved on their own in a few days. On the 3rd of March 2021, she received her first dose of the ChAdOx19 (Recombinant) vaccination [COVISHIELD]. The following day she experienced generalised myalgia. Ten days after vaccination, she developed difficulty walking and noticed re-emergence tremors in both legs below the knee. On the 21st of April, she received her second dose of the vaccine. The next day she developed pain and a burning sensation in her lower limbs associated with urinary and faecal incontinence. She was admitted to a nearby hospital.

**Fig. 4:**
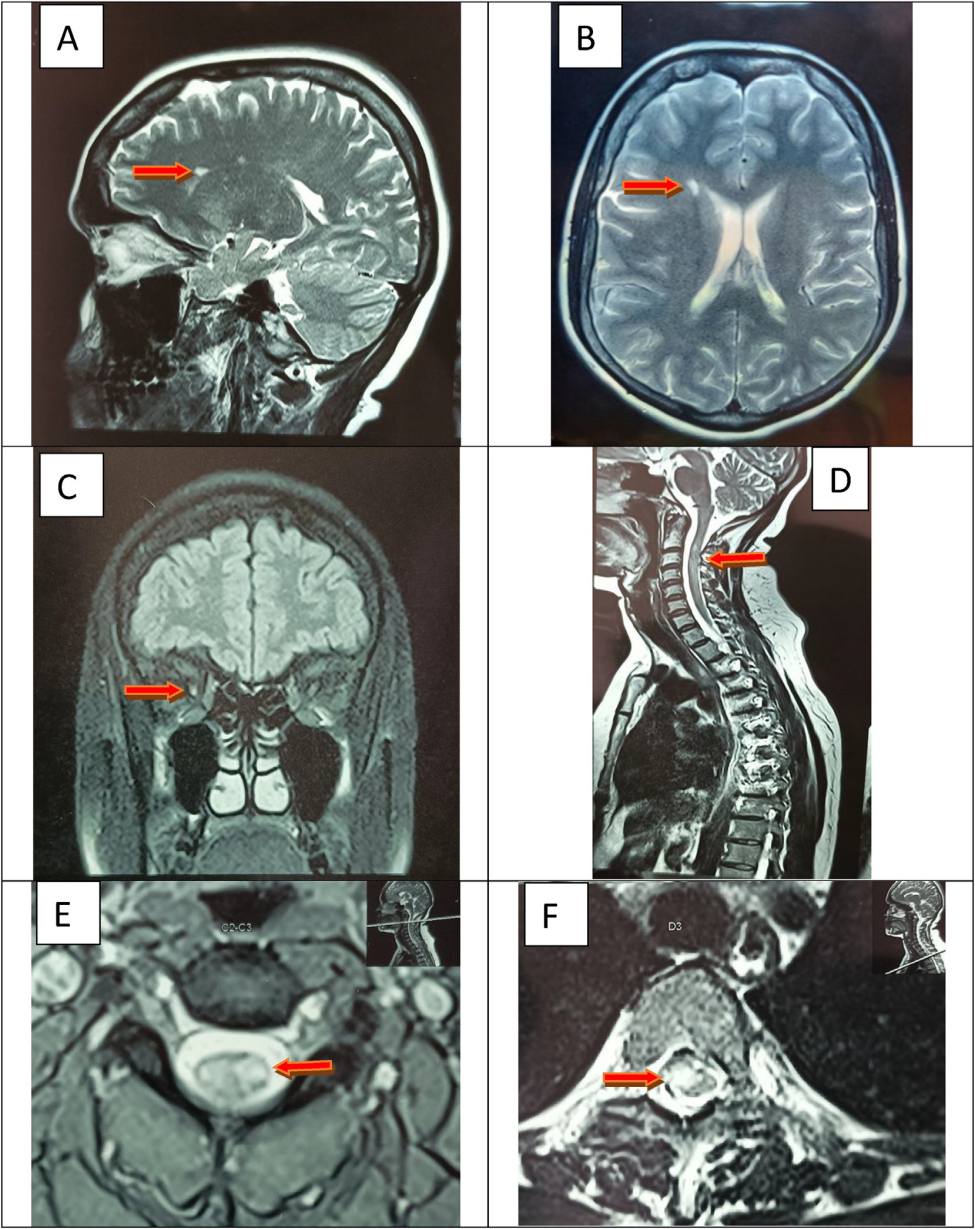
Brain magnetic resonance imaging (MRI) findings. Few hyperintense periventricular lesions were observed in T2 weighted images (A: Sagittal image & B: Axial image). Hyperintense signals in the right optic nerve on T2 weighted image (C: Coronal image). Hyperintense lesions in the C1, C2 & C3 on the left side (D: Sagittal image & E: Axial image) and D3 on the right side on T2 weighted image (F: Axial image).

MRI of the Cervical-Dorsal spine plain and contrast detected hyper-intense lesions in the C1, C2, and C3 on the left side and C4/D3 on the right side on T2 weighted images, which were iso-intense in T1 weighted images which were suggestive of sub-acute demyelination. MRI brain revealed few hyperintense periventricular lesions on T2 weighted images, which were iso-intense in T1 weighted images and showed no diffusion restriction or contrast enhancement suggestive of sub-acute or older demyelination. Also, there were hyperintense signals in the right optic nerve on T2 weighted images, which could have been normal signal variation or sub-acute neuritis [Fig. 4]. Myelin Oligodendrocyte Glycoprotein (MOG) and NeuromyelitisOptica (NMO) IgG antibodies were negative [Supl file 4]. CSF study for oligoclonal bands noted the presence of 15 unique oligoclonal bands. The multiple Sclerosis evaluation panel supported the diagnosis of Multiple Sclerosis [Suppl File 5]. Six months before treatment at NARIP, the patient was on a Disease-modifying drug (Dimethyl Fumarate) and other medications for controlling neuropathic pain (Ritala 354 mg & Gabapentin) & anxiety (Duloxetine HCL & Clonazepam).

Initially, the patient was administered IVMP 1 gm OD for five days. The patient reported minimal progress during a hospital stay. She was discharged with advice to take oral prednisolone for seven days. Meanwhile, on the 28th of May, she tested positive for COVID infection, following which she stopped all medicines as per medical advice. She restarted oral prednisolone on the 12th of June. After one week, Dimethyl fumarate 240 mg (Disease Modifying Drug – DMD) twice a day was advised. Symptoms persisted. On the 27th of November, she was admitted to NARIP, presenting with complaints of paraesthesia in lower limbs, arthralgia, walking difficulties, and urinary and faecal incontinence.

#### 1.2.2 Clinical findings

On examination, tenderness and restricted movements due to pain were observed in the right elbow, right knee, right ankle, and left hip. Romberg's test was positive, showing unsteady tandem walking (swaying towards the right side). Somato-sensory sensations were normal. She demonstrated hyperreflexia in deep tendon reflexes at the biceps-triceps bilaterally and at the left knee. The Kurtzke Expanded Disability Status Scale (EDSS) and the Functional Assessment of MS (FAMS) yielded scores of 5 and 138, respectively, on admission.

#### 1.2.3 Timeline

**Fig. 5:**
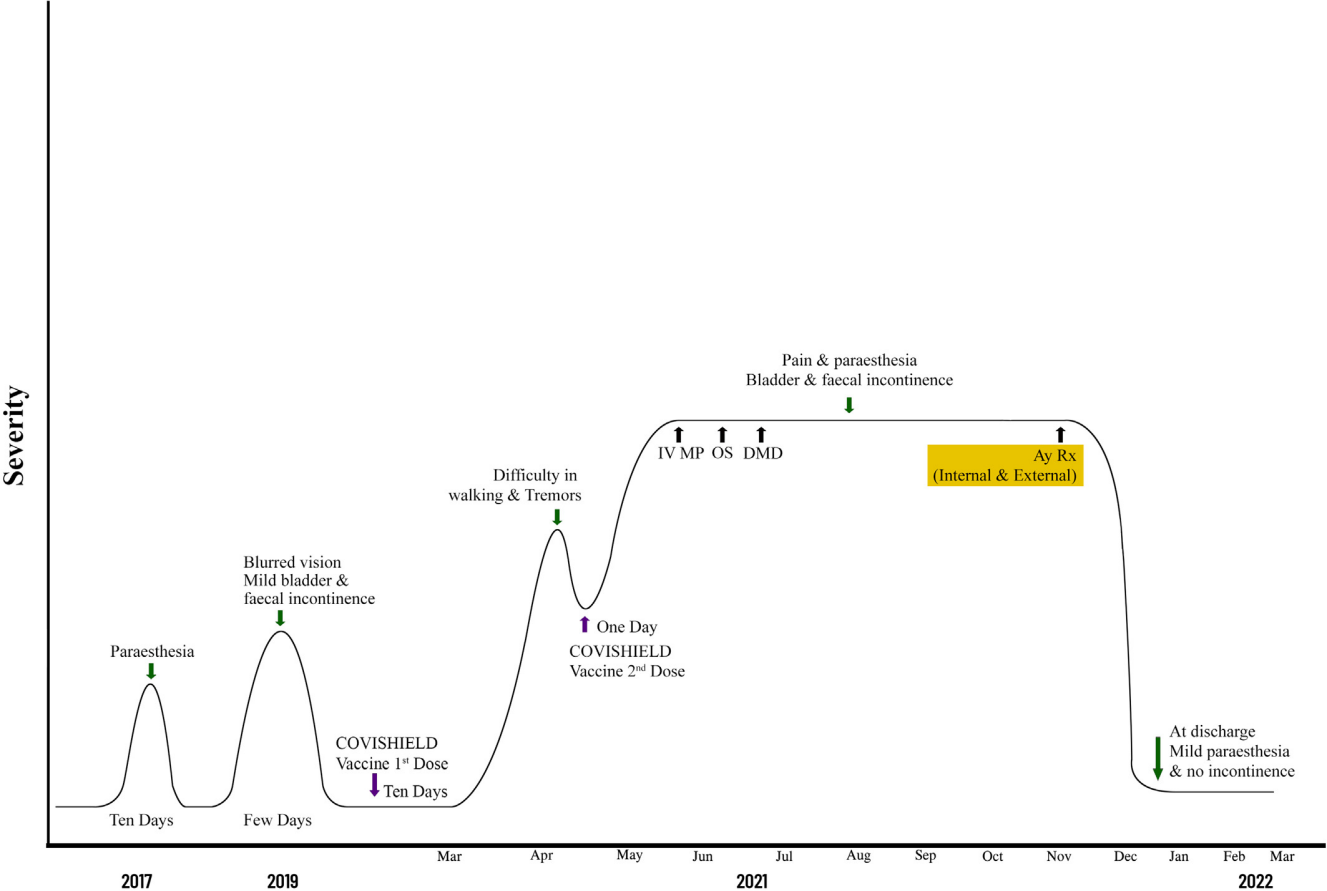
Clinical course of the condition of Case 02 demonstrating the rapid progression and exacerbation of symptoms after two doses of Covid-19 vaccination. The patient had two episodes of mild symptoms, which drastically progressed after the Covid-19 vaccination. The condition remained status quo even after the usage of steroids and DMD. Further, the patient had substantial improvement after a short course of Ayurvedic treatments, and the improvement was consistent during the follow-up period of 3 months. Abbreviations: IV-MP, Intravenous Methylprednisolone; OS, Oral Steroids; DMD, Disease Modifying Drugs; Ay TRT, Ayurvedic Treatment.

Fig. 5 corresponds to the graphical representation of the clinical manifestation of MS in the patient, its speed progression, exacerbation before and after Covid-19 vaccination, and the clinical improvement in the condition after Ayurvedic management. The first notable symptom of paresthesia was reported in 2017, which lasted about ten days and later subsided. Then after two years, i.e., in 2019, symptoms viz. blurred vision and impairment in bladder control developed. These symptoms also subsided within a few days. However, a drastic change in the disease progression pattern was noticed after the first dose of the COVISHIELD vaccine in February 2021. The patient developed severe symptoms like difficulty in walking and tremors. These symptoms aggravated after the second dose of the same vaccine in April 2021. The patient developed pain, paresthesia, bladder, and faecal incontinence also. Following this episode, she was diagnosed with MS. The patient responded to Ayurvedic treatments after a short course of treatment for one month and got discharged from the hospital in February 2022 with relatively milder symptoms. From the graphical note, it is noticeable that from a relapsing-remitting course of MS, the patient was heading towards a secondary progressive variant of the disease. [21]. Ayurvedic treatment, along with DMD, effectively managed the symptoms in the patient, and the imrovement in the condition was consistent during the follow-up period of 3 months.

#### 1.2.4 Therapeutic intervention

She was administered internal medications and external therapies until the 13th of January 2022 [Table 1, Table 2].

#### 1.2.5 Follow-up and outcomes

The Kurtzke Expanded Disability Status Scale (EDSS) and the Functional Assessment of MS (FAMS) yielded scores of 1 and 91, respectively, on discharge. At the time of discharge, mild paraesthesia & occasional arthralgia persisted, while there were no walking difficulties anymore. Also, urinary and faecal incontinence disappeared. The condition was consistent during the follow-up period of 3 months.

## 2 Discussion

Inflammation-driven autoimmune pathways can specifically disturb nervous system physiology [22]. Of the many causes triggering autoimmune reactions in the nervous system, infections play a candidate role [23]. Although uncommon, vaccine-induced inflammatory responses of CNS are not unheard of [24]. CNS demyelinating conditions following vaccination have been reported [25].

The world witnessed much research fund allocation on the COVID-19 outbreak's epidemiology, pathology, impact on lifestyles, social behaviours and treatment possibilities [[26], [27], [28], [29], [30], [31]]. The highly contagious nature of the disease has compelled scientific communities and related organisations to hasten vaccine development and supplies. Well-timed international collaborations have resulted in quicker development of varied forms of vaccines against COVID-19 [32]. Scientists and clinicians have acknowledged the need for caution while the COVID vaccination campaign globally, especially when whole communities (comprising of elderly, children, other vulnerable populations, populations with cardiovascular or neurological disorders or other comorbidities, immune-compromised individuals or individuals on immunotherapy) are to be inoculated [10]. Prospective observational studies and systematic reviews on vaccine trials have reported their safety and efficacies [33,34].

Nevertheless, post-marketing surveillance is quintessential to ascertain such safety and efficacy claims. Several adverse temporal events, such as haematological, immunological and neurological untoward occurrences, have been reported following COVID-19 inoculation [5,6,12,[35], [36], [37]]. There is growing evidence of the impact of COVID vaccination in patients with neurological-neuroimmunological disorders. Immune mechanisms and related physiological processes are complicated and, most importantly, personalised. Therefore causal association between the vaccine and such adverse events remains putative. Retrodictive analysis and subsequent reporting of individual events shall give a more vivid picture to determine the nature and extent of causality in such conditions that can ultimately have a significant and cumulative impact on public health.

Management of inflammation-driven autoimmune disorders is challenging. Preventing relapses or aiming at long-term remission through a significant decrease in symptoms and improved quality of life are achievable goals in Acute Demyelination (AD) management [38]. Host responses and subsequent auto-immunity are categorically personalised. Hence, a standard treatment protocol in all ADs may sometimes prove irrelevant. Addressing aggressive immune responses with single-handed immune suppression strategies, especially in inflammation-driven autoimmune nervous system pathologies such as MS and ADEM, seems to need to be more comprehensive. Owing to its heterogeneities in causative factors, pathogenic circuits, and expression of symptoms, a comprehensive treatment strategy is essential for the complete remission of ADs such as MS and ADEM. The effectiveness of current treatment strategies of symptom-based approaches or immune suppression protocols highly depends on the stage of the disease. Such treatment protocols seem less adaptable in chronic inflammation or secondary progression phases in respective autoimmune conditions [38].

Inflammation-driven demyelination and related neurological symptomatology can be understood in Ayurvedic parlance across varied contexts, for instance, Vatarakta, Avaritavata, Sannipatajwara, Dhathugatajwara, and Dhathukshaya [Table 3]. Therefore Ayurvedic clinical practice guidelines in such conditions should be contextually decoded and formulated. Analysing stage-wise presentation of either condition, ADEM in the particular patient apparently co-related to kaphaavruta/kaphaanubandha vyana-udanavayu sanga/samajwara anubandha vatavyadhi states; whereas MS presentation by its nature of relapse-remission traits significantly resembled vatarakta samprapthi. Treatments administered were based on these clinical co-relations. The proposed mode of action of medicaments and treatments are concisely depicted in table no 1 & 2. Treatment protocol administered here plausibly manages inflammation driven neurological symptoms (jwarachikitsa, vatarakta chikitsa and avruta vata chikitsa) and enhances neuro-protective/rehabilitative measures (vatavyadhi chikitsa / with special mention to bruhmana in later stages).

**Table 3:** Ayurvedic parlance for corresponding neurological symptomatology of Demyelinating diseases.

Symptoms in both cases	Symptoms in the patient	Ayurvedic understanding of the symptomatology						
		Dosha involved^a^	Doshakopa/Dhathukshaya^b^	Sannipathajwara^c^	Dhathugatajwara^d^	Vatavyadhi^e^	Vatarakta^f^	Avaraitavata^g^
**Case 01 (ADEM)**								
Sensory symptoms (loss of sensation/paresthesia/dysesthesia)	Paresthesia	PranaVayu VyanaVayu	✓	✓	✓	✓	✓	✓ (Kapha avrita vata)
Motor symptoms (weakness/tremor/spasticity/fatigue)	Weakness, Tremors, Fatigue Arthralgia/LBA	UdanaVayu PranaVayu	✓	✓	✓	✓	✓	✓ (Kapha avrita Udana)
Cortical signs (aphasia, agraphia, alexia)	Agraphia	Prana vayu Udana vayu	✓	–	–	✓	–	✓ (Kapha avrita Udana)
Bulbar symptoms (dysarthria/dysphagia)	Dysarthria	Udana vayu Prana vayu	✓	–	–	✓	–	✓ (Kapha avrita Udana)
Vestibular symptoms (vertigo/gait balance)	Gait abnormalities – unsteady gait, Ataxic gait	VyanaVayu PranaVayu	✓	✓	✓	✓	✓	✓ (Kapha avrita Vyana)
Cognitive symptoms (memory impairment, impairment of executive functions, trouble concentrating)	Memory loss	UdanaVayu PranaVayu	✓	✓	–	–	–	–
Psychiatric symptoms (depression, anxiety)	Emotional disturbances	PranaVayu UdanaVayu	✓	✓	–	–	–	–
**Case 02 (MS)**								
Sensory symptoms (loss of sensation/paresthesia/dysesthesia)	Paresthesia	PranaVayu VyanaVayu	✓	✓	✓	✓	✓	✓ (Pitta avrita vyana)
Motor symptoms (weakness/tremor/spasticity/fatigue)	Tremors, Fatigue Arthralgia	UdanaVayu PranaVayu	✓	✓	✓	✓	✓	✓ (Pitta avrita vyana)
Vision symptoms (optic neuritis/double vision/vision loss)	Blurred vision	PranaVayu Chakshur vaisheshikaAlochaka pitta	✓	–	✓	✓	–	–
Urinary and bowel symptoms (incontinence, retention, urgency, constipation, diarrhoea, reflux)	Urinary and faecal incontinence	ApanaVayu Samana Vayu	✓	✓	–	✓	–	–
Altered balance	Walking difficulties	VyanaVayu PranaVayu	✓	✓	✓	✓	✓	✓ (Pitta avrita vyana)

a AstangaHridaya, Sootra sthana, Chapter 12.

b Caraka Samhita, Sootra sthana, Chapter 17/Susrutha Samhita Sootra sthana, Chapter 15/AstangaHridaya, Sootra sthana, Chapter 11.

c Susrutha Samhita Uttara tantra, Chapter 39/Caraka Samhita, Chikitsa sthana, Chapter 3/Madhava Nidana, Chapter 2.

d Susrutha Samhita Uttara tantra, Chapter 39/Caraka Samhita Nidana sthana, Chapter 1/Madhava Nidana, Chapter 2.

e AstangaHridayaNidana sthana, Chapter 15.

f&g Astanga Hridaya Nidana sthana, Chapter 16.

The reported two cases of demyelinating conditions of post-COVID-19 vaccination sequel were managed effectively with Ayurvedic internal & external treatment procedures. There was considerable improvement in neurological deficits in both cases. Substantial improvement was noted in the follow-up MRI brain done in ADEM. The clinical status remained almost normal during the follow-up period in both cases. Further, it was observed that Ayurvedic treatments and DMD helped maintain the condition of MS in the remission phase consistently. However, either cases accounted for different set of confounders and heterogeneity. Hence well planned clinical trials are necessary to formulate justifiable hypotheses.

## 3 Conclusion

The holistic disease-modifying model is effective in managing the progression of demyelinating diseases. Though it is presumed that the benefit of vaccination outweighs the risk of neurological deficits post-vaccination in predisposed autoimmune neurological conditions, such events can adversely affect daily activities and quality of life. Hence, a medical panel should thoroughly evaluate the fitness of individuals with existing diagnosed or suspicious neurological symptoms and those on disease modifiers that suppress immune mechanisms.

It is believed that case series and case reports shall judiciously add information to vaccine safety data and acknowledge the necessity of clinician approval based on individualised assessments before vaccination. Moreover, these reports also lead to holistic care treatment models incorporating Traditional Medicine (TM) in neurological autoimmune diseases that comprehensively manage inflammation and bring evident clinical/symptomatological relief in patients. Laboratory and brain-imaging evidence following TM administration should also be congregated to ascertain the disease-modifying effects of such protocols.

## Patient's perspective

The perspective is added as supplementary files.

## Informed consent

Informed consent has been received from patients and is added as supplementary files.

## Author contribution

Pratap Shankar K.M. did the clinical aspects of the study, while Pratibha P Nair prepared and presented the work. Saritha V gave inputs on radiological findings.

## Funding

This research received no specific grant from public, commercial, or not-for-profit funding agencies.

## Conflicts of interest

None.

## Figures and Tables

**figs1:**
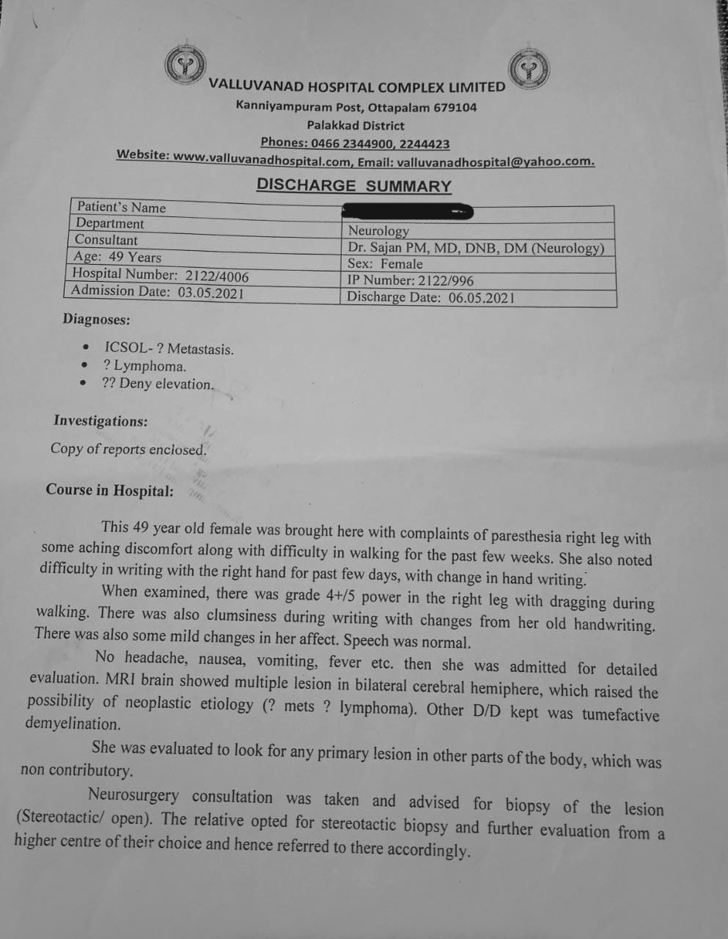


**figs2:**
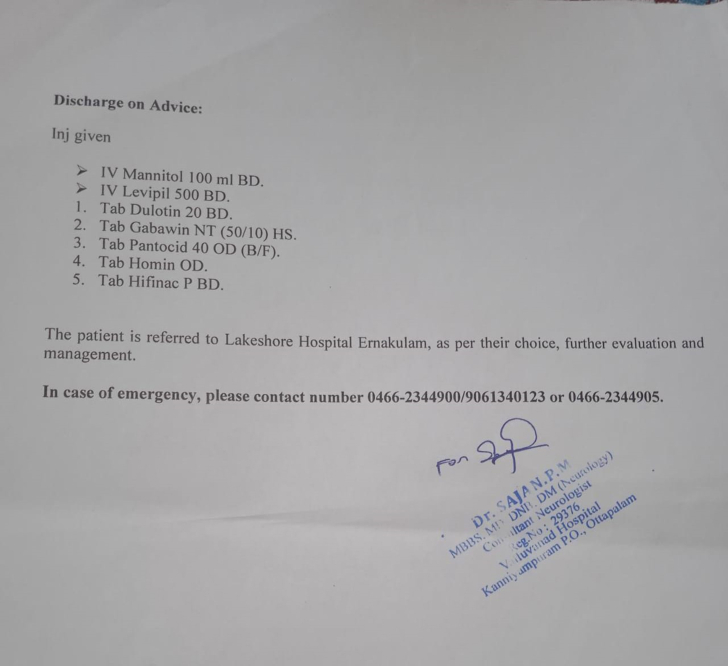


**figs3:**