# Supplementary material for: Ayurvedic management of neurological deficits post COVID-19 vaccination - A report of two cases
Source: J Ayurveda Integr Med. 2023 Jun 8;14(3):100737. doi: 10.1016/j.jaim.2023.100737 (PMC10247886; doi:10.1016/j.jaim.2023.100737)
Supplement: Multimedia component 1 [file mmc1.pdf]

Printed Date:28/05/2021 16:38:24

NEURO IMMUNOLOGY LABORATORY SERVICE REPORT

Patient Name: Mrs. [redacted] **MRD#:** 2273896
Date of birth: 07/05/1973 **Sex:** Female
Age: 48Y 21D **Date:** 27/05/2021

Service Order:
Multiple Sclerosis Evaluation Panel
Neuroimmunology Laboratory Service Report Reference No-17375/2021/Vol-25
Client patient ID:498012
Ref by Dr. Vidhya M.V, HOD , Dept of Neurology, Lakeshore Hospital, Kochi.

Interpretation:
Methodology: Oligoclonal band assay by Isoelectric Focusing and immunofixation of CSF and serum.

Albumin Serum:4.3 g/dL Ref value:3.7 to 5.2 g/dL

IgG Serum :1442.0 mg/dL Ref value: 700 to 1600 mg/dL

Albumin CSF:17.80 mg/dL Ref value: 8 to 42 mg /dL

CSF IgG:2.67 mg/dL Ref value: 0.8 to 7.7 mg/dL

CSF IgG index:0.45 Ref value: less than or equal to 0.66

CSF IgG Synthesis Rate: 5.54 mg/24hr Ref value:less than or equal to 8mg/24hr

Albumin quotient: 0.41% Ref Value : Normal upto 0.7 %, Mildly positive upto 2.0 %, Moderately positive upto 5.0 %, Severely positive more than 5.0 %

Albumin index: 4.13 Ref Value :<9.0 no significant impairement of BBB
9.0-14.3 Slight impairment;14.4-33.3-moderate impairement;
33.4-100- Severe impairment;>100.0-Total breakdown
Intrathecal IgG - <0.0 mg% Ref Value:<0.0 mg/dL

Serum Oligoclonal Bands : No bands detected
CSF Oligoclonal Bands : No bands detected

This test showed no bands in CSF and serum.

Reference Value; 0 to 3 bands

Comments:
This test showed normal CSF IgG index , normal CSF IgG synthesis rate, and normal IgG Intrathecal synthesis.Absence of Oligoclonal band in CSF suggests that there is no intrathecal IgG synthesis and this report is not consistent with Multiple Sclerosis.

Normal Albumin index indicates no impairment of blood brain barrier.

Reference:

Hans Link,Yu-Min Huang:Oligoclonal bands in Multiple Sclerosis cerebrospinal fluid; An update on methodology and clinical usefulness. J Neuroimmunol, 2006;180;17-28

Ziadie M, Wians Jr FH. A guide to the interpretation of CSF indices. Laboratory Medicine. 2005 Sep 1;36(9):558-62.

Signed By: Dr. Sudheeran Kannoth
