# Supplementary material for: Ayurvedic management of neurological deficits post COVID-19 vaccination - A report of two cases
Source: J Ayurveda Integr Med. 2023 Jun 8;14(3):100737. doi: 10.1016/j.jaim.2023.100737 (PMC10247886; doi:10.1016/j.jaim.2023.100737)
Supplement: Multimedia component 2 [file mmc2.pdf]

Printed Date:24/05/2021 17:46:35

NEURO IMMUNOLOGY LABORATORY SERVICE REPORT

Patient Name: [redacted]
MRD#: 2276474
Date of birth: 23/05/1983
Sex: Female
Home Phone: 952664-5043
Age: 38Y 2D
Date: 24/05/2021

Service Order:
Neuromyelitis Optica Spectrum Disorders (NMOSD) screen for the detection of autoantibodies to Myelin Oligodendrocyte Glycoprotein (MOG) and Neuromyelitis Optica (NMO/Aqp4)
Neuroimmunology Laboratory Service Report Reference No-17434/2021/Vol-26
Client patient ID:2122/9783
Ref by Ref by Dr.V.T Ravi, Consultant Neurologist/ Dr.Ameen Amarakkadan, Dept of Critical Care, Moulana Hospital, Peerinthalmanna, Malappuram.

Interpretation:
Serum sample tested negative for MOG IgG antibody and negative for NMO IgG antibody by indirect immunofluorescence on transfected cells.

Comments:
NMOSD is a clinical diagnosis. A negative test does not exclude the diagnosis of NMO.If strongly suspected, repeat examination after 3 months is recommended.

MOG IgG antibody is often associated with seronegative NMOSD and other demyelinating disorders.

Reference:
Lennon VA,Kryzer T.J,Pittock S.J etal,JExp Med 202 (2005) 473-77
Franciotta D, Gastaldi M, Sala A etal. Diagnostics of the neuromyelitis optica spectrum disorders (NMOSD);Neurol Sci. 2017 Oct;38(Suppl 2):231-236.

Signed By: Dr. Sudheeran Kannoth
