# Supplementary material for: Ayurvedic management of neurological deficits post COVID-19 vaccination - A report of two cases
Source: J Ayurveda Integr Med. 2023 Jun 8;14(3):100737. doi: 10.1016/j.jaim.2023.100737 (PMC10247886; doi:10.1016/j.jaim.2023.100737)
Supplement: Multimedia component 3 [file mmc3.pdf]

Printed Date:02/06/2021 17:06:47

NEURO IMMUNOLOGY LABORATORY SERVICE REPORT

Patient Name: [redacted]
MRD#: 2276474
Date of birth: 23/05/1983
Sex: Female
Home Phone: 952664-5043
Age: 38Y 11D
Date: 02/06/2021

Service Order:
Multiple Sclerosis Evaluation Panel
Neuroimmunology Laboratory Service Report Reference No-17434/2021/Vol-26
Client patient ID:2122/9783
Ref by Ref by Dr.V.T Ravi, Consultant Neurologist/ Dr.Ameen Amarakkadan, Dept of Critical Care, Moulana Hospital, Peerinthalmanna, Malappuram.

Interpretation:
Methodology: Oligoclonal band assay by Isoelectric Focusing and immunofixation of CSF and serum.

Albumin Serum: 4.8 g/dL Ref value:3.7 to 5.2 g/dL

IgG Serum : 1132.0 mg/dL Ref value: 700 to 1600 mg/dL

Albumin CSF:14.40 mg/dL Ref value: 8 to 42 mg /dL

CSF IgG:9.12 mg/dL Ref value: 0.8 to 7.7 mg/dL

CSF IgG index: **2.68** Ref value: less than or equal to 0.66

CSF IgG Synthesis Rate: **29.64 mg/24hr** Ref value:less than or equal to 8mg/24hr

Albumin quotient: 0.3% Ref Value : Normal upto 0.7 %, Mildly positive upto 2.0 %, Moderately positive upto 5.0 %, Severely positive more than 5.0 %

Albumin index: 3 Ref Value :<9.0 no significant impairement of BBB
9.0-14.3 Slight impairment;14.4-33.3-moderate impairement;
33.4-100- Severe impairment;>100.0-Total breakdown

Intrathecal IgG **- 6.72 mg%** Ref Value:<0.0 mg/dL

Serum Oligoclonal Bands : No bands detected
CSF Oligoclonal Bands : **15 bands** detected (unique)

This test showed **15 bands** in CSF and no band in serum.

Reference Value; 0 to 3 bands

Comments:
An elevated CSF IgG index, elevated IgG synthesis , elevated IgG local synthesis and the presence of **15 unique** oligoclonal bands in CSF indicates intrathecal synthesis of IgG.Four or more bands supports the diagnosis of Multiple Sclerosis.

Normal Albumin index indicates no impairment of blood brain barrier**.**

Reference:

Hans Link,Yu-Min Huang:Oligoclonal bands in Multiple Sclerosis cerebrospinal fluid; An update on methodology and clinical usefulness. J Neuroimmunol, 2006;180;17-28

Ziadie M, Wians Jr FH. A guide to the interpretation of CSF indices. Laboratory Medicine. 2005 Sep 1;36(9):558-62.

Signed By: Dr. Sudheeran Kannoth
